# Late gadolinium enhancement and T2 MR imaging features of cardiac sarcoidosis involving the left and right ventricle

**DOI:** 10.1186/1532-429X-11-S1-P92

**Published:** 2009-01-28

**Authors:** Varghese Cherian, Hersh Chandarana, Ruth P Lim, Leon Axel, Danny Kim, Monvadi B Srichai

**Affiliations:** NYU Langone Medical Center, New York, NY USA

**Keywords:** Right Ventricular, Sarcoidosis, Cardiac Magnetic Resonance, Late Gadolinium Enhancement, Cardiac Sarcoidosis

## Objective

To evaluate the presence and location of late gadolinium enhancement (LGE), increased T2 signal abnormality, right ventricular involvement and cardiac function by cardiac magnetic resonance (CMR) imaging in patients with suspected cardiac sarcoidosis.

## Materials and methods

46 patients with known extra-cardiac sarcoidosis underwent CMR with T2-weighted, LGE and cine imaging for evaluation of cardiac sarcoidosis. Images were evaluated by 2 expert reviewers in consensus using the 17-segment model of the left ventricle (LV) for presence and location (subepicardial, midwall, subendocardial or transmural) of LGE, presence of increased T2-signal abnormalities, and regional wall motion abnormalities. In addition, right ventricular (RV) involvement was evaluated.

## Results

13 of 46 (28%) patients demonstrated evidence of segmental LGE suggestive of cardiac sarcoidosis. In these 13 patients, LGE involvement was noted in 51 of 221 (23%) segments, and was predominantly transmural (35%), followed by midwall (27%), subepicardial (25%) and subendocardial (20%). LGE was most frequently located in the basal segments (36%) compared to mid (23%) and apical (22%) segments, and more commonly involved the septal (37%) followed by the lateral (29%), inferior (23%) and anterior (10%) segments. T2-weighted images were evaluable in 11 of the 13 patients, and 24 of 187 segments (13%) demonstrated evidence of increased T2-signal abnormality and was significantly associated with the LGE segmental involvement (p < 0.005). 4 patients (31%) also demonstrated evidence of RV involvement. There was no significant correlation between presence of LGE or increased T2-signal intensity with regional function.

## Conclusion

Late gadolinium enhancement and increased T2-signal intensity are common findings in the left ventricle in patients with cardiac sarcoidosis, but not necessarily correlated with overall ventricular size and function. Although not previously reported, similar findings are frequently present in the right ventricle as well.

